# Review of Transport Phenomena and Popular Modelling Approaches in Membrane Distillation

**DOI:** 10.3390/membranes11020122

**Published:** 2021-02-08

**Authors:** Yan Dong, Xiaodong Dai, Lianyu Zhao, Li Gao, Zongli Xie, Jianhua Zhang

**Affiliations:** 1 Department of Oil Engineering, Shengli College China University of Petroleum, Dongying 257061, China; 000158@slcupc.edu.cn (Y.D.); xiaodongdai1980@163.com (X.D.); 2 YunFu (Foshan) R&D Center of Hydrogen Energy Standardization, Yunfu 527326, China; lyzhao@alum.imr.ac.cn; 3 South East Water Corporation, P.O. Box 2268, Seaford, VIC 3198, Australia; Li.Gao@sew.com.au; 4 CSIRO Manufacturing, Private Bag 10, Clayton South MDC, VIC 3169, Australia; zongli.xie@csiro.au; 5 Institute for Sustainable Industries & Liveable Cities, Victoria University, Melbourne, VIC 8001, Australia

**Keywords:** membrane distillation, mass transfer, heat transfer, modelling

## Abstract

In this paper, the transport phenomena in four common membrane distillation (MD) configurations and three popular modelling approaches are introduced. The mechanism of heat transfer on the feed side of all configurations are the same but are distinctive from each other from the membrane interface to the bulk permeate in each configuration. Based on the features of MD configurations, the mechanisms of mass and heat transfers for four configurations are reviewed together from the bulk feed to the membrane interface on the permeate but reviewed separately from the interface to the bulk permeate. Since the temperature polarisation coefficient cannot be used to quantify the driving force polarisation in Sweeping Gas MD and Vacuum MD, the rate of driving force polarisation is proposed in this paper. The three popular modelling approaches introduced are modelling by conventional methods, computational fluid dynamics (CFD) and response surface methodology (RSM), which are based on classic transport mechanism, computer science and mathematical statistics, respectively. The default assumptions, area for applications, advantages and disadvantages of those modelling approaches are summarised. Assessment and comparison were also conducted based on the review. Since there are only a couple of full-scale plants operating worldwide, the modelling of operational cost of MD was only briefly reviewed. Gaps and future studies were also proposed based on the current research trends, such as the emergence of new membranes, which possess the characteristics of selectivity, anti-wetting, multilayer and incorporation of inorganic particles.

## 1. Introduction

Membrane technology has been widely studied in recent years for purification and separation. Membrane distillation (MD), as an emerging technology, is a membrane-based separation process involving liquid–vapour phase change. Although the mass transfer in the MD is incurred by the vapour pressure difference, thermal energy is required to drive the mass transferring across the membrane. MD was firstly introduced in the late 1960s [1] and has been attracting more interest in the research area since the 1980s due to the advances of the new membrane materials and the promoted comprehension of the principles being associated to mass and heat transfers of MD [2]. On 5 May, 1986, at the Workshop on Membrane Distillation, Rome, MD was formally recognised and defined by a “Round Table Meeting” [3]. In MD systems, the major function of the feed/permeate pumps is not for driving mass through the membrane, but to provide adequate turbulence and to mitigate the concentration and temperature polarisations associated with the mass transfer across the membrane. The hydrophobic microporous membranes for MD only allow vapours to transfer from the feed side to the permeate side, which is driven by the effective vapour pressure difference being incurred by temperature difference and/or reduced pressure between the feed and permeate sides. Therefore, the driving force of MD would not be influenced greatly by the feed concentration as the membrane processes need to overcome the osmosis pressure such as from nanofiltration and the reverse osmosis process. In comparison with the hydraulic pressure/potential -riven membrane separation processes and technologies used in desalination, MD has some distinctive advantages, such as a theoretically 100% rejection of components that are non-volatile, low pumping pressure, large membrane pore size, compact vaporisation space compared to conventional distillation (Multi-Stage Flash Distillation) and mild feed vaporisation temperature (40–80 °C) [4,5]. Thus, theoretically, MD is able to utilise low-grade heat energy to treat high salinity water with a relatively small footprint.

MD systems are generally divided into four major configurations based on the methods being used to apply a low vapour pressure on the permeate side [6]:

(A) Direct contact MD (DCMD), in which the cold liquid/permeate directly contacting the membrane is used to generate the temperature difference [7,8].
(B) Air gap MD (AGMD), in which a low-temperature air gap is on the permeate side and interposed between the membrane and a condensation surface cooled normally by a cooling flow [9].
(C) Vacuum MD (VMD), in which the reduced pressure created by a vacuum pump or other devices is applied on the permeate side to generate the vapour pressure difference [10,11].
(D) Sweep gas MD (SGMD), in which a sweeping gas is used on the permeate side to stripe vapour transferred from the feed side [12,13,14,15].

Although the mechanism of the mass and heat transfer varies in different configurations, enthalpy of vaporization/condensation (latent heat) is required for phase change from liquid to gas in all configurations [16]. Hence, a significant amount of thermal energy needs to be supplied for mass transfer in MD. Even considering that “free” heat sources are available in MD application, the energy consumed by pumps to circulate the feed/permeate through the module is also considerable in comparison with other membrane processes, since high flow turbulence is the key to maintain both high flux and high thermal energy efficiency. However, for some reason, the energy consumption of the pumps is generally ignored in total energy efficiency calculation.

Commercial applications for membrane distillation were generally considered in the area where other processes are hard to achieve satisfying results [17,18,19]. Beijing China Science Resources and Environmental Technology Co. Ltd. (CSRE) has successfully developed and commercialized a vacuum multi-effect membrane distillation (V-MEMD) process for corrosive wastewater treatment [20]. It was found from the commercialization of the V-MEMD that the comprehensive cost of an MD plant using high-grade heat (steam) is actually lower than utilising low-grade heat source (80 °C) hot water, in which the cost of overall operation efficiency and treatment capability (m^3^/h) are included. Lower operation efficiency or lower treatment capability will lead to an increase in the fixed cost for per unit volume of treated water and consume more pumping energy to circulate the feed, which is hardly included in any modelling program currently.

Hence, modelling is a significant tool for the commercialisation of MD to assess the feasibility of utilising MD to treat the given wastewater.

Many modelling approaches have been employed in the membrane distillation:

- Conventional modelling approaches [21], which are based on the foundation of mass transfer and heat transfer through porous materials. Characterisation of the membrane and flow dynamics are critical to providing acceptable modelling results. Some membrane properties obtained prior to the tests could be varied during the experiment under different pressures and temperatures and need to be fitted with the operation conditions in the modelling accordingly [22].
- Computer simulation approaches, such as computational fluid dynamics (CFD). CFD models were developed in the early 1950s, initially for solving aeronautic problems [23]. They have been widely used as an analysis tool to study transport phenomena in MD [24]. Membrane characteristics and flow dynamic information are still required for the simulation. However, CFD can be used to study the hydrodynamic conditions, heat and mass transfers in MD modules for optimisation purpose [25,26].
- Mathematical statics approaches, such as response surface methodology (RSM) [27,28,29]. RSM is a collection of mathematical and statistical methods based on fitting a polynomial equation to the experimental data and is able to predict the behaviour of a data set with the objective of making statistical previsions [30].

From modelling, the membrane flux can be predicted, and the membrane area required under the given operation conditions can be estimated. The modelling could also provide information for membrane module design and optimise the operation conditions [24,31]. These days, with the assistance of computer science, membrane design or structure can also be optimised based on the targets through modelling [32].

There are many review papers published on MD modelling. Olatunji et al. [33] conducted a comprehensive review of the modelling of mass and heat in MD configuration. Hitsov et al. [21] made a critical review of different approaches in MD modelling, in which different models were summarised. However, there are hardly any reviews providing a summary of assumptions for purposes of simplifying the modelling approaches of different configurations, which were sometimes neglected or noticed by the researchers. This ignorance could hinder the error analysis and possibly amplify the errors when using the models under certain conditions. Therefore, a summary of assumptions for each approach is reviewed and presented. Cost modelling is essential for commercialisation and was generally conducted based on “free” thermal energy and even a “free” pumping energy, generally ignoring investment cost. A brief review of the costing modelling emphasizes the importance of involving those factors in the cost modelling. Many researchers have misused temperature polarisation to assess thermal efficiency, which is clarified in this paper.

## 2. Mechanism of Heat Transfer and Mass Transfer through Porous MD Membranes

**Figure 1:**
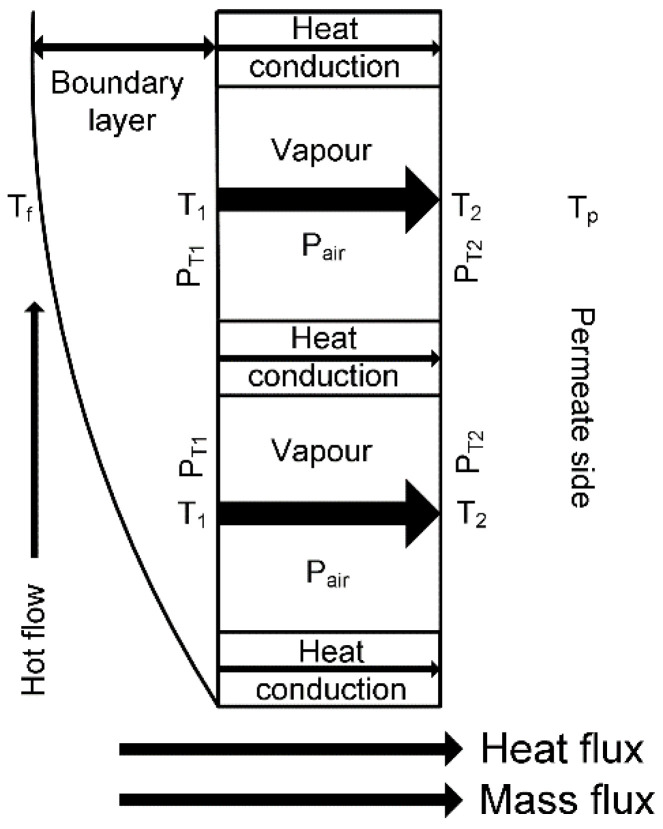
Heat transfer and mass transfer through membrane.

In MD, heat and mass transfers occur simultaneously and are in the same direction from the hot side to the cold side [34], as shown in Figure 1. The feed temperature drops from *T_f_* across the feed side boundary layer to *T*_1_ at the membrane surface across the feed side boundary layer on the feed side. Feed water evaporates at the membrane surface and transfers through the porous membrane with latent heat. Simultaneously, heat is conducted through the porous membrane from the feed side to the permeate side. The heat conduction and latent heat transfer result in the bulk temperature of the permeate *T_p_* increasing to *T*_2_ at the membrane surface. The driving force is, therefore, the vapour pressure difference between the vapour pressure *P_T_*_1_ of *T*_1_ and vapour pressure *P_T_*_2_ at the membrane surface on the permeate side and is less than the difference between vapour pressure at *T_f_* and vapour pressure calculated with the permeate bulk temperature *T_p_* in DCMD and AGMD. This phenomenon is called temperature polarisation (TP).

### 2.1. Heat Transfer from the Feed Stream to the Permeate Side

Heat transfer through the feed side to the permeate side includes two steps [35]:

- Firstly, heat transfers from the feed side to the permeate side across the porous membrane as sensible heat and latent heat, which incur the temperature difference between the boundary layer and bulk flow;
- Secondly, the temperature difference between the bulk flow and membrane surface leads to heat transfers from the bulk flow to the boundary layer via heat convection.

Hence, the heat balance of the feed can be expressed by [34,36].

(1) $$Q_{1}=\frac{\lambda }{b}\left(T_{1}-T_{2}\right)+JH_{latent}$$

(2) $$\lambda =\lambda_{air}\varepsilon +\lambda_{solid}\left(1-\varepsilon \right)$$

(3) $$Q_{2}=\alpha_{f}\left(T_{f}-T_{1}\right)=Q_{1}$$

(4) $$\alpha_{f}\left(T_{f}-T_{1}\right)\;=\frac{\lambda }{b}\left(T_{1}-T_{2}\right)+JH_{latent}$$

where *Q*_1_ and *Q*_2_ are the total heat transferred from the feed side to the permeate side; *λ*, *λ_air_* and *λ_solid_* are the thermal conductivities of the membrane, air and membrane material; *b*, *ε* and *A* are, respectively, thickness, porosity and area of the membrane; *α_f_* is the convective heat transfer coefficient; and *J* and *H_latent_* are the flux and latent heat of vapour, respectively.

In Equation (1), the sensible heat conducted through the membrane is (*λ/b*)*A*(*T*_1_ − *T*_2_), and *JH_latent_* is the thermal energy used for mass transfer/evaporation. In MD operation, it is desirable to minimise the sensible heat loss or maximise the heat for evaporation. To lower the conduction of sensible heat, it could increase the membrane porosity as shown in Equation (2) to reduce the heat transfer coefficient (*λ/b*), since the thermal conductivity of the membrane materials is generally one order of magnitude greater than that of the air. To enhance mass transfer, it would be preferable to increase *T*_1_ [37,38]. To achieve this, it is necessary to boost the convective heat transfer coefficient based on Equations (1), (3) and (4). The convective heat transfer coefficient can be calculated as [39]

(5) $$\alpha_{f}=-\frac{\lambda_{f}}{T_{f}-T_{1}}{\left(\frac{dT}{dy}\right)}_{boundary}$$

where *λ_f_* is thermal conductivity of the feed and ${\left(\frac{dT}{dy}\right)}_{boundary}$ is the temperature gradient perpendicular to the membrane surface in the thermal boundary layer as shown in Figure 1 on the feed side.

In Equation (5), it can be found that reducing the difference of (*T_f_* − *T*_1_) and/or increasing the temperature gradient could increase the convective heat transfer coefficient, which can be realised by thinning the thermal boundary layer. Enhancement of the turbulence of the fluid is able to reduce the thickness of the thermal boundary layer. Turbulence promoters can effectively reduce the thickness of the thermal boundary layer and improve *α_f_* [40,41] but cause a low-pressure drop in the channel, where the feed solution and/or cooling liquid are flowing [37,38,42,43]. It was reported that the temperature polarisation coefficient (*TPC*) defined by Equation (6) of spacer filled channels is in the range of 0.9–0.97, in comparison with 0.57–0.76 in the channels without a spacer [37].

(6) $$\tau =\frac{T_{1}-T_{2}}{T_{f}-T_{p}}$$

where *τ* is the temperature polarisation coefficient.

Reynolds number of the spacer-filled channel can be expressed by [37,43].

(7) $$Re=\frac{\rho v_{s}d_{h}}{\mu }$$

where *v_s_* is the linear velocity of the fluid, *ρ* and *μ* are the density and viscosity of the fluid and *d_h_* is the hydraulic diameter of the flow channel. The linear velocity *v_s_* can be calculated by [43]

(8) $$v_{s}=\frac{Q_{f}}{A_{cross}\varepsilon_{spacer}}$$

where *Q_f_* is volumetric flow rate of the fluid, *ε_spacer_* is the spacer porosity and *A_cross_* is the area of the channel cross-section.

The hydraulic diameter of the channel *d_h_* can be calculated by [40]

(9) $$d_{h}=\frac{4\;\varepsilon_{spacer}}{\left(\frac{2}{h_{sp}}\right)+\left(1-\varepsilon_{spacer}\right)S_{spacer}}$$

where *h*_sp_ and *S_spacer_* are the thickness and the specific surface of the spacer, respectively and *S_spacer_* can be expressed by

(10) $$S_{spacer}=\frac{4}{d_{f}}$$

where *d_f_* is the filament diameter of the spacers. The spacer porosity can be measured experimentally or calculated by

(11) $$\varepsilon_{spacer}=1-\frac{\pi d_{f}^{2}}{2l_{m}h_{sp}sin\theta }$$

where *l_m_* is the mesh size.

Based on the above discussion, it is important to use a highly porous membrane and enhance the fluid turbulence to increase proportions of the thermal energy for mass transfer.

### 2.2. Mass Transfer through the Porous Membrane

Three steps are involved in the MD mass transfer:

- Firstly, vaporisation of feed at the liquid/gas interface or the interface between the membrane and the feed;
- secondly, transportation of vapour from the interface between the feed and the membrane to the interface between the membrane and permeate side, which is driven by the vapour pressure difference across the membrane pores; and
- thirdly, transportation of the vapour from the interface between the membrane and permeate side into the permeate side [44].

For the first step, the control factors include the evaporation temperature (interface temperature) and the evaporation area (surface porosity), which are exponential and linear relationships to mass transfer, respectively [45]. The control factors for the second step are the mean length and area of the membrane matrix for vapour transport, which are related to the pore size, porosity, pore tortuosity and thickness of the membrane. The third step is controlled by the vapour pressure and pore area (surface porosity) at the interface between the membrane and the permeate side. In those factors, the surface porosity and the mean length and area of the membrane matrix for vapour transport are attributed to the membrane properties, and the evaporation temperature and vapour pressure at the pore interface are determined by the operation conditions. Both of the membrane properties and operation condition such as availability of the heat sources could be the limiting step for the mass transfer [46].

The transportation of gas phase through the porous membrane is normally dominated by three theoretical principles: Knudsen-diffusion (*K*), Poiseuille-flow (*P*) and Molecular-diffusion (*M*) or a transition mechanism between them [35,47]. For a given MD process, the dominant principle of mass transfer through the membrane pores is determined by a Knudsen number (*Kn*), defined in Equation (12).

*Kn* = *l*/*d* (12)

where *d* is the mean diameter of the pores and *l* is the mean free molecule path, which can be calculated by [48,49]

(13) $$l=\frac{k_{B}T}{\pi {\left((\sigma_{w}+\sigma_{a})/2\right)}^{2}P_{pore}}\frac{1}{\sqrt{1+\left(m_{w}/m_{a}\right)}}$$

where *k_B_* is the Boltzman constant (1.381 × 10^−^^23^ JK^−1^), *σ_w_* and *σ_a_* are the collision diameters of the water molecule (2.641 × 10^−^^10^ m) and air (3.711 × 10^−^^10^ m) [50], *T* is the mean temperature in the pores and *m_w_* and *m_a_* are the molecular weights of water and air.

At a normal operation temperatures of MD (40–60 °C), the mean free molecule path of the water is approximately 0.1 µm. Since the pore sizes of the MD membranes used are generally in the range of 0.1 to 1.0 μm, *Kn* will be in the range of 0.1 to 1. In DCMD, AGMD and SGMD, it is gas mixture (air and water) in the pores, and the total pressure difference equals zero. Based on Table 1 [46], it can be found that Knudsen-diffusion is the dominant mass transfer principle in DCMD < AGMD and SGMD. In VMD, it can be considered that a single gas (water vapour) is in the pore, since the air will be replaced by the water vapour under total pressure difference. Therefore, the Poiseuille flow and Knudsen diffusion contribute to the mass transfer in VMD.

**Figure 2:**
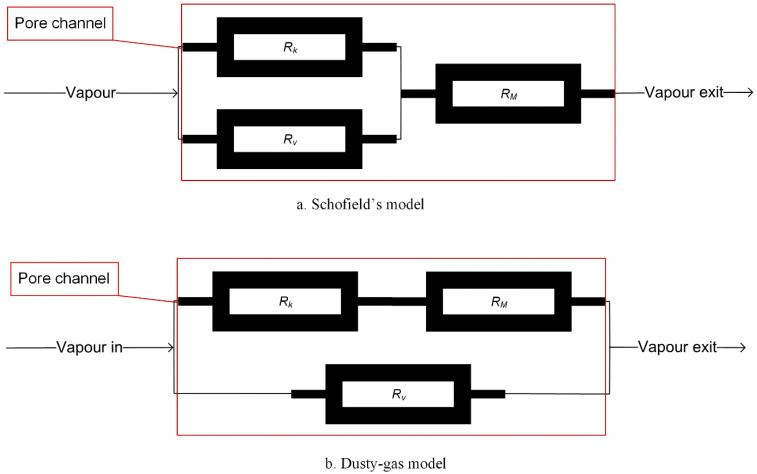
Gas transport mechanisms in electrical circuit analogues (*R_K_* is the resistance to Knudsen-diffusion, *R_M_* is the resistance to Molecular-diffusion, *R_V_* is the resistance to viscous flow, (**a**). Schofield’s model; (**b**). Dusty gas model).

An electrical circuit analogue can be used to describe the transport mechanism of the gas molecule through the porous membrane [6,52], as shown in Figure 2, in which Schofield’s model [36] as shown in Figure 2a and the dusty-gas model, as shown in Figure 2b [53,54], are the other two most popular models used in MD.

Developed from kinetic theory, the Schofield’s model (Figure 2a) assumes that the total permeability is the sum of Knudsen permeability and viscous permeability, in which the viscous flow would be ignored if it is a gas mixture in the pore. When the single gas is in the pores, the transition region between the Knudsen and viscous flows can be described by

$N=-M\bar{v}\left(A+B\frac{\sqrt{2}\sigma P_{pore}}{k_{B}T}\right)\frac{\left(P_{T1}-P_{T2}\right)}{b}$, in which

(14) $$A=\frac{d\varepsilon }{3tRT},\;\mathrm{and}\;B=\frac{\pi r^{2}\varepsilon }{128RT}$$

where *P_T_*_1_ and *P_T_*_2_ are vapour pressures at membrane interfaces, respectively, on feed and permeate sides, $\bar{v}$ the gas’ mean molecular speed, *b* is the membrane thickness, *P_pore_* is the pressure in the pore and *σ* is the cross-section of collision.

In the Dusty-Gas model, the matrix of the porous membrane is assumed to be an array of dust particles, which are viewed as one component of the gas mixture but fixed in the space [53]. In this model, the gas flux passing through the membrane in the Knudsen-viscous transition region is described as

$J=-\frac{M}{RT}\left[(K_{0}\bar{v}+\frac{B_{0}P_{pore}}{{µ}_{g}}\right)\frac{\left(P_{T1}-P_{T2}\right)}{b}]$, in which

(15) $$K_{0}=\frac{d\varepsilon }{3\tau },\;\mathrm{and}\;B_{0}=\frac{\varepsilon d^{2}}{32\tau }$$

where *µ_g_* is the viscosity of the gas.

In those models, the mean pore size is generally used and could possibly incur significant error due to the wide distribution of the pore size, since the mass transfer mechanism varies with the pore size [55]. For example, as shown in Table 1, when the pore size is 2 µm, the mass transfer mechanism is M, P or M-P, but when the pore size is 0.01 µm, the mass transfer mechanism is K. Assuming that 95% of pore size is 0.01 µm and 5% of pore size is 2 µm, the mean pore size is 0.11 µm. Therefore, the transfer mechanisms are in the transition area based on the mean pore size. If the mean pore size is used in the model, it would underestimate the membrane resistance to mass transfer, since the dominant mechanism is K based on the percentage of the pore size. Woods et al. [56] made a comparison between the models with and without considerations of the pore size distribution and found for mean pore sizes of a membrane in range of 0.2 to 0.45 μm, membrane pore size distribution would have a relative influence on the modelling prediction compared to the experimental results.

### 2.3. Interaction of Heat and Mass Transfers

Since the MD is associated with evaporation, both thermal energy and mass are transferred simultaneously from the feed side to the permeate side as shown in Figure 1. In Equation (1), the latent heat is the minimum thermal energy required for a single-stage MD to drive mass from the feed to the permeate side. Thermal conduction is considered as heat loss, which compromises the thermal efficiency defined in Equation (16) [44,57].

(16) $$E=\frac{JH_{latent}\;}{\Delta Q_{f}}\%=\frac{JH_{latent}\;}{C_{f}{\dot{m}}_{f}\left(T_{f,i}-T_{f,o}\right)}\%$$

where *E* is the thermal efficiency; ∆*Q_f_* is the total heat loss from the feed flow; and *C_f_*, *ṁ_f_*, *T_f,i_* and *T_f,o_* are the specific heat, mass flow rate of the feed, and inlet and outlet temperatures of feed, respectively.

The thermal efficiency is normally higher than 80% [17,58] in DCMD when the feed temperature is greater than 60 °C. For similar conditions, AGMD and SGMD could achieve a thermal efficiency greater than 90% [59,60], and VMD is able to achieve a thermal efficiency higher than 95% [61]. By suppressing the thermal conduction loss, it can increase the thermal efficiency effectively. Since the thermal conduction loss is a linear relation in comparison with the exponential relation of latent heat transfer to the temperature difference across the membrane, increasing the feed temperature and/or permeate temperature would enhance the thermal efficiency [59,60]. Similarly, enhancing the flow turbulence will also reduce the difference between *T_f_* and *T*_1_, as shown in Figure 1, and promotes the thermal efficiency by enlarging the temperature difference across the membrane.

Although high temperature would increase the thermal efficiency, it would also enhance the difference between *T_f_* and *T*_1_ or temperature polarisation under the same hydrodynamic conditions. The convective heat transfer coefficient described in Equation (5) would not vary greatly if the hydrodynamic conditions maintain the same. When the temperature on the feed side increases, both the latent heat transfer and sensible heat transfer become greater. If the variation of the convective heat transfer coefficient is negligible, it can be found from Equation (4) that the difference of (*T_f_* − *T*_1_) or temperature polarisation will become greater. Thus, the temperature polarisation is not directly related to high flux or high thermal efficiency. From Equation (4), it is clearly shown that the larger temperature polarisation will facilitate the higher flux when the convective heat transfer coefficient (*α_f_*) is constant. Therefore, it can be concluded that the heat transfer resistance (1/*α_f_*) directly affects the flux and thermal efficiency. Similarly, increasing the linear velocity of the feed will increase both the flux (*J*) and total energy transfer (*α_f_*(*T_f_* − *T*_1_)) from the feed side to the permeate side but reduce the temperature polarisation/temperature difference between the bulky feed and membrane surface (*T_f_* − *T*_1_) [31,58,61]. Therefore, the increment in magnitude of the convective heat transfer coefficient should be greater than the decrease in magnitude of the temperature difference to enable the increment of the total thermal energy transfer. It can also be found that when (*T_f_* − *T*_1_) is approaching zero, to maintain effective mass transfer, *α_f_* needs to approach infinity, which is practically impossible due to high pumping energy consumption and the limitation of the hydraulic pressure applied on the MD membrane. Hence, temperature polarization is not a very good parameter to assess the MD process.

For a single-stage MD, even if the thermal efficiency is 100%, the energy consumption is huge in comparison to other membrane processes. For example, at a feed inlet temperature of 60 °C and permeate inlet temperature of 20 °C, the energy consumption is 655 kWh/m^3^ [62] solely for driving the distillate across the membrane, which does not include the cooling energy to maintain the permeate at 20 °C and pumping energy to maintain adequate flow turbulence. However, the energy consumption of RO, multiple stage flash and multiple effect distillation are, respectively, 13 to 36 kWh/m^3^ [63], 92.5 kWh/m^3^ and 73 kWh/m^3^ [64]. Although “low-grade” heat could be used in MD, the percentage of pumping energy consumption will increase significantly due to low flux if the feed temperature is lower than 40 °C based on the observation from our previous study [65].

Multiple stage design could lower specific thermal energy in membrane distillation significantly. Vacuum multi-effect MD, multistage liquid gap MD and multistage AGMD are able to practically reuse the latent heat up to 3.2 times (gain and output ratio). When the stage number of VMD increases to 20 [66], the latent heat can be reused up to more than 4 times, in which the vacuum is applied gradually at each stage and the vapour is condensed by the feed post evaporation at a lower temperature to recover the latent heat. Although the reuse times of latent heat is still lower than the reported 9.5 times achieved by the multiple stage flash [67], free low-grade heat in combination with low maintenance and investment costs makes MD attractive for water production in remote areas.

### 2.4. Mass Transfer and Heat Transfer in Four Major Configurations

In the four major configurations, the heat transfer phenomena are the same on the feed side but are different on the permeate side. The mass transfer from the feed across the membrane and into the permeate side is also different in these configurations.

#### 2.4.1. Direct Contact Membrane Distillation

**Figure 3:**
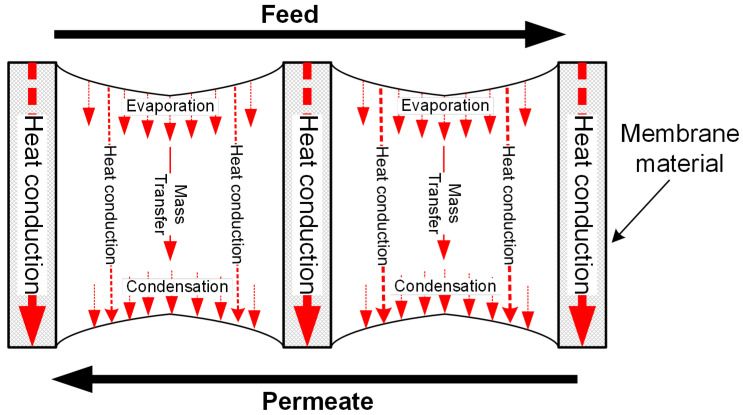
Schematic of heat and mass transfers in direct contact membrane distillation (DCMD).

In Figure 3, the schematic of heat and mass transfers in DCMD is shown. In the membrane pores, it is an air-vapour mixture and there is no total pressure difference. Assuming the pore size distribution width is less 1.2 and *Kn* is in the range of 0.1–1, based on Table 1, the mass transfer through the membrane pore is a Knudsen-molecular diffusion transition mechanism and can be expressed as [52,56]

$$\frac{1}{J}=\frac{1}{J_{M}}+\frac{1}{J_{K}}$$

in which,

(17) $$J_{K}=\frac{4}{3}d\frac{\varepsilon }{bt}\sqrt{\frac{m_{w}}{2\pi RT}}\left(P_{T1}-P_{T2}\right),\;\mathrm{and}\;J_{M}=\frac{m_{w}}{1-x_{A}}\frac{\varepsilon D_{AB}}{btRT}\left(P_{T1}-P_{T2}\right)$$

where *J_M_* and *J_K_* are the mass transfer contributed from molecular and Knudsen diffusions respectively, *t* is tortuosity of the pore channel, *R* is the universal gas constant [68] and *D_AB_* (m^2^/s) is the relative diffusivity of water vapour (A) to air (B) and can be estimated in the temperature range of 273–373 K by [35,41,69],

(18) $$D_{AB}=\frac{1.895\times 10^{-5}T^{2.072}}{P}$$

In DCMD, on the permeate side, the membrane directly contacts the liquid phase, which would conduct the heat from the feed side into the bulk stream quickly and decrease the temperature at the interface between the membrane and the permeate. Hence, the driving force of DCMD is relatively great in all four configurations. However, the large temperature difference also enhances heat conduction loss as shown in Equation (1), but it would not be necessary to reduce the thermal efficiency described in Equation (16), since the latent heat increases exponentially with temperature. Only in the range where the temperature change incurs less change rate of vapour pressure than *λ*/*b*, the temperature decline at the interface of the permeate side will lower the thermal efficiency [20].

In comparison with other MD configurations, DCMD has the highest proportion of heat conduction loss through the membrane. Furthermore, since the vapour condenses directly into the permeate stream, it is not easy to recover the latent heat from the permeate stream by a multistage design, which has limited gained output ratio (can also be defined by Equation (16)) to less than 1 [70,71].

#### 2.4.2. Air Gap Membrane Distillation

**Figure 4:**
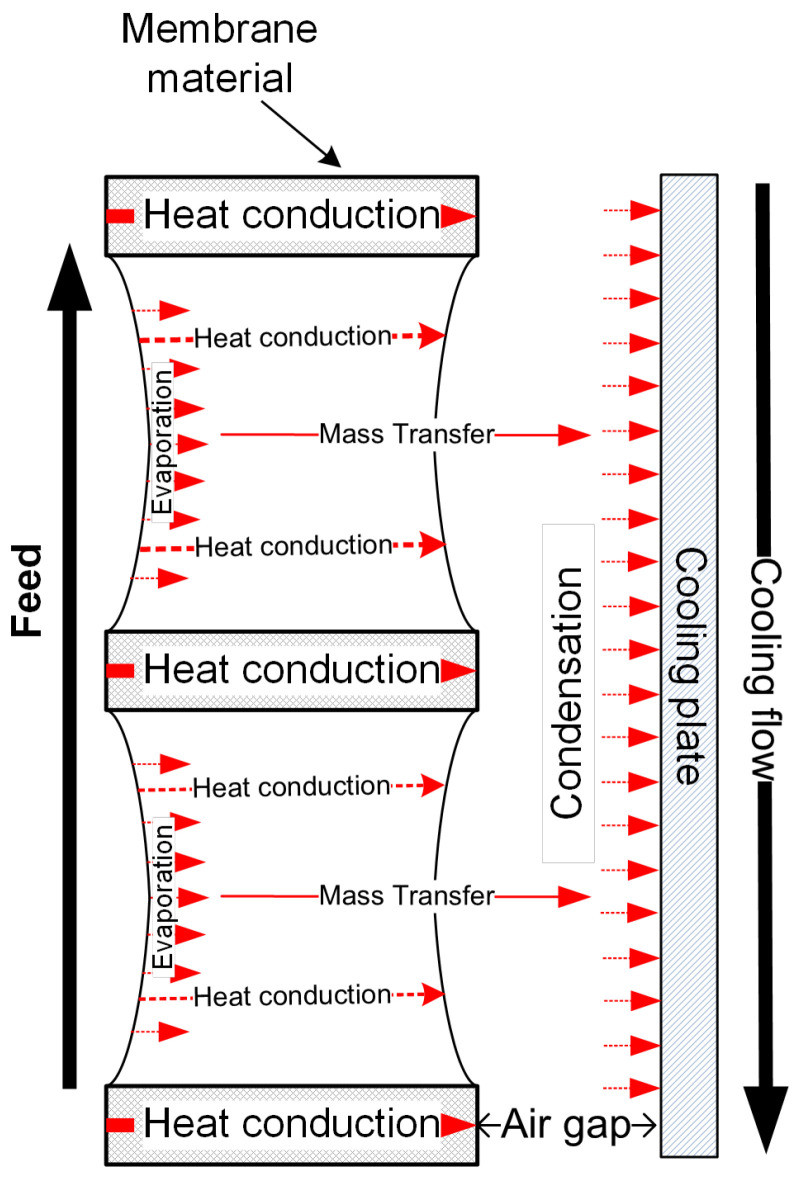
Schematic of heat and mass transfers in air gap membrane distillation (AGMD).

The schematic of heat and mass transfer is shown in Figure 4. Similar to DCMD, in the membrane pores, it is also an air-vapour mixture, and there is no total pressure difference across the pore. Therefore, the mass transfer through the membrane pore in AGMD is also a Knudsen-molecular diffusion transition mechanism. However, the vapour also has to pass through the air gap prior to the condensation on the membrane surface. The mass transfer in the air gap of AGMD can be calculated by [72]:

(19) $$J=M\frac{P_{g}D_{AB}}{RT_{g}}\left(x_{2}-x_{c}\right)$$

where *P_g_* and *T_g_* are the mean pressure and temperature in the air gap and *x*_2_ and *x_c_* are the molar fractions of the water vapour at surfaces of membrane and condensate, respectively.

The heat transfer in AGMD on the permeate side is different from the DCMD since it is an air gap. The evaporated water from the membrane surface on the permeate side will not release its latent heat until it condenses on a cooling plate [73]. Since the membrane surface on the permeate side is not directly in contact with the cooling flow, *T*_2_ is higher than that of DCMD under the same feed operation conditions, and the conduction loss in AGMD is less than DCMD based on Equation (1). Since in AGMD, an equilibrium state of air and water vapour is generally assumed in the membrane pores [73,74], the driving force of AGMD is also the temperature difference (*T*_1_ − *T*_2_) between both sides of the membrane. Hence, the mass transfer driving force in AGMD is less than that of DCMD under the same feed conditions.

The heat transfer on the permeate side of the membrane in AGMD includes:

- Heat transfer (*Q_gap_*) through the air gap can be described by:

(20) $$Q_{gap}=\frac{\lambda_{air}}{b_{air}}\left(T_{2}-T_{c}\right)+JH_{latent}$$

where *T_c_* is the surface temperature of the condensate and *b_air_* is the thickness of the air gap.

- Heat transfer (*Q_c_*) through the condensate layer, where the temperature decreases from *T_c_* to *T_pl_*:

(21) $$Q_{c}=\frac{\lambda_{c}}{b_{c}}\left(T_{c}-T_{pl}\right)+JH_{latent}$$

where *T_pl_* is the temperature of interface between the condensate and the cooling plate, and *λ_c_* and *b_c_* are the thermal conductivity and the thickness of the condensate layer, respectively.

- Heat transfer (*Q_pl_*) through the cooling plate:

(22) $$Q_{pl}=\frac{\lambda_{pl}}{b_{pl}}\left(T_{pl}-T_{cl}\right)$$

where *T_cl_* is the temperature of interface between the cooling plate and the cooling flow, and *λ_pl_* and *b_pl_* are the thermal conductivity and the thickness of the cooling plate, respectively.

- Heat transfer (*Q_cl_*) through the cooling flow:

(23) $$Q_{cl}=\alpha \left(T_{cl}-T_{cl,b}\right)$$

where *T_cl,b_* is the temperature of the bulky cooling flow, and α is the convective heat transfer coefficient.

Under the stable mode, in the AGMD,

(24) $$Q_{1}=Q_{gap}=Q_{c}=Q_{pl}=Q_{cl}$$

The *TPC* in AGMD can be defined as

(25) $$\tau =\frac{T_{1}-T_{2}}{T_{f}-T_{cl,b}}$$

#### 2.4.3. Sweeping Gas Membrane Distillation

**Figure 5:**
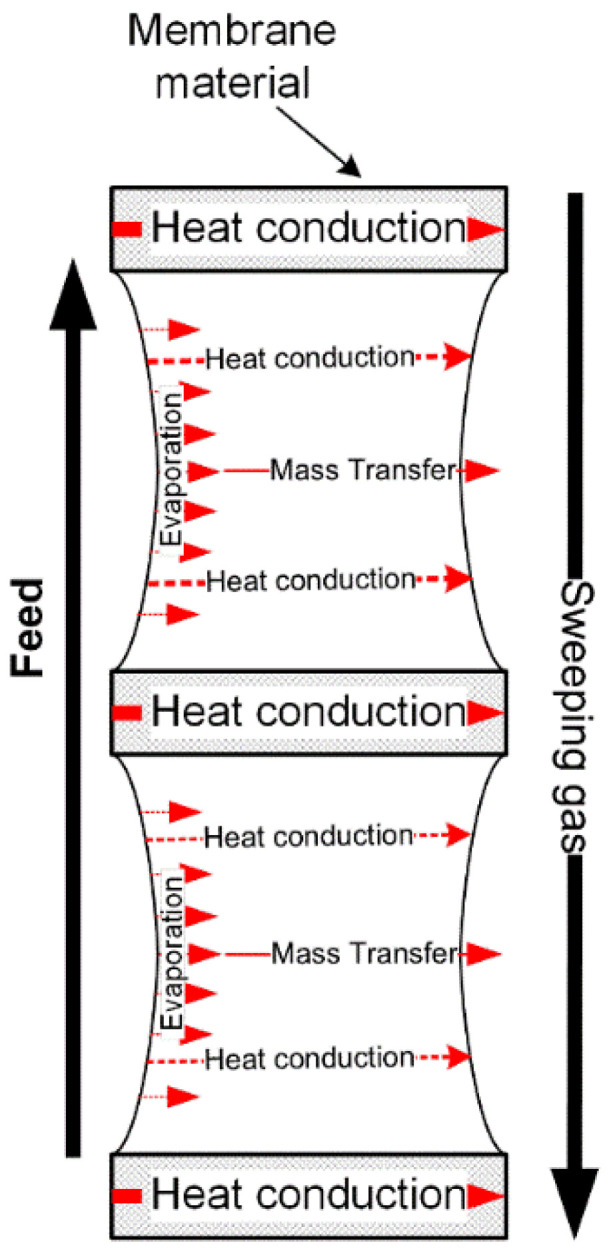
Schematic of heat and mass transfers in sweep gas membrane distillation (SGMD).

As shown in Figure 5, the permeate side of SGMD is a moving gas instead of the liquid flow in DCMD or static air in AGMD. However, there is no difference between SGMD and AGMD/DCMD, in the aspect of the mass transfer mechanism through the membrane pores, as shown in Table 1.

The vapour transfer from the membrane surface on the permeate side to the bulk gas flow depends on the humidity and velocity of the gas [75] and can also be expressed by Equation (19). Compared with the static air layer in AGMD, the gas stream would greatly enhance the mass transfer. *x_c_* of the sweeping gas is much less than that of AGMD, since the fresh air flow has lower relative humidity and higher vapour diffusion enhanced by high flow turbulence than of the static air gap. Therefore, with the same feed conditions, the flux of SGMD should be greater than that of the AGMD. However, condensation and re-evaporation could occur during the operation due to the temperature change in the sweeping gas chamber and possible humidity change of the sweeping gas [76,77] and will affect the mass transfer in the SGMD.

The heat transfer on the feed side and through the membrane pores of the SGMD are the same as DCMD and AGMD. On the permeate side of the membrane, the heat transfer of SGMD can be expressed as

(26) $$Q_{sg}=h_{sg}\left(T_{2}-T_{sg}\right)+JH_{latent}$$

where *Q_sg_* is heat transfer from the membrane surface on the permeate side to the bulk gas stream, *h_sg_* is the convective heat transfer coefficient and *T_sg_* is the temperature of the bulk gas flow.

In Equation (26), *T_sg_* is not directly related to the driving force. As long as the *x*_2_ is greater than *x_c_*, theoretically, there should be mass transfer from the feed to the sweeping gas stream, even if *T_sg_* is greater than *T*_2_ or *T_f_*. Hence, in SGMD, *TPC* cannot be used to assess the polarisation of the driving force. The real driving force for mass transfer in SGMD is the difference between the vapour pressure at *T*_1_ on the feed side and vapour pressure linear to *x*_2_ at the membrane interface on the permeate side. However, the apparent mass transfer driving force of SGMD is the difference between the vapour pressure at *T_f_* on the feed side and vapour pressure linear to *x_c_* of the bulk flow of the sweeping gas. It can be found from Equations (3) and (19) that *T_f_* and *x*_2_ are greater than *T*_1_ and *x_c_*. Therefore, the rate of driving force polarisation (*τ_p_*) can be expressed by

(27) $$\tau_{p}=\frac{P_{T1}-x_{c}P}{P_{Tf}-x_{2}P}$$

where *P* is the total pressure of the sweeping gas.

#### 2.4.4. Vacuum Membrane Distillation

**Figure 6:**
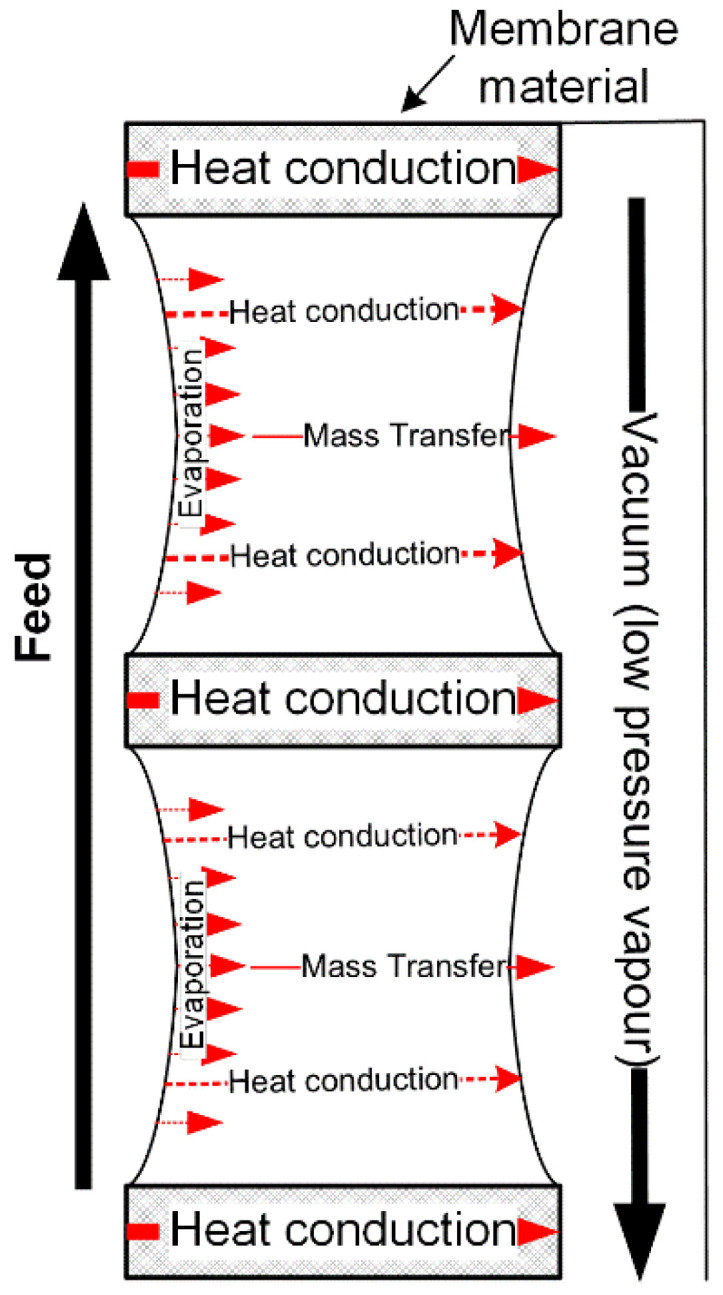
Schematic of heat and mass transfers in vacuum membrane distillation (VMD).

As shown in Figure 6, it is single gas in the membrane pores, and the mass transfer driving force in VMD is the total pressure difference between the saturated vapour at membrane interface on the feed side and the vacuum pressure in the permeate chamber, which is distinctive from all other MD configurations. In VMD, it can be assumed that only water vapour is in the pores. Therefore, based on Table 1, the Poiseuille-flow–Knudsen-diffusion transition mechanism (*P*–*K*) is the dominant mass transfer mechanism of VMD. Since both of Poiseuille flow and Knudsen diffusion contribute to the mass transfer, the flux of VMD can be described by

(28) $$J=J_{K}+J_{P}=\left(\frac{8}{3}\frac{r\varepsilon }{\tau b}\sqrt{\frac{1}{2\pi RMT}}+\frac{\varepsilon r^{2}}{\tau b}\frac{1}{8\eta }\frac{P_{pore}}{RT}\right)\left(P_{T1}-P_{vacuum}\right)$$

where *P_vacuum_* is the absolute pressure in the vacuum chamber.

In VMD, since the permeate side of VMD is under reduced pressure, the heat conduction loss from the membrane to the permeate side can be ignored [35,61]. The heat transfer on the feed side of VMD is the same as that of other MD configurations. On the permeate side, the heat transfer can be expressed as

(29) $$Q_{vacuum}=JH_{latent}$$

where *Q_vacuum_* is the heat transfer on the permeate side.

Similar to the SGMD, it is not feasible to use temperature polarisation for the assessment of the driving force polarisation in VMD, but the rate of driving force polarisation (*τ_p_*) can be used:

(30) $$\tau_{p}=\frac{P_{T1}-P_{vacuum}}{P_{Tf}-P_{vacuum}}$$

#### 2.4.5. Comparison of Thermal Energy Efficiency in Four Configurations

In Table 2, flux and thermal efficiency of the single-stage MD reported from previous studies are shown. It can be found that those data varied greatly, because the research studies were conducted under very different conditions, such as materials and configuration of membrane, feed characteristics, temperatures, fluid velocities and module configuration.

Based on Equation (16), assuming the feed heat losses in all four configurations are the same, it can be found that VMD should have the highest thermal energy efficiency, which is close to 100% since there is negligible thermal conduction loss. The permeate sides of both AGMD and SGMD are gas phase, but SGMD would have higher flux [78] and have comparable or lower thermal efficiency to AGMD [79]. The permeate side of DCMD is the liquid phase. Therefore, thermal conduction loss from DCMD is the highest, which leads to the lowest thermal efficiency in the four configurations.

#### 2.4.6. Separation Organics and Water Mixtures by Hybrid Membrane

MD is not commonly used in separation of organic/water mixture, since the hydrophobic MD membrane could be easily wetted by the organic solvents and lose its separation efficiency [90,91]. Furthermore, the separation factor between organics and water in MD is limited by the relative volatility defined by Raoult’s law as shown in Equation (31) [92].

(31) $$\alpha_{i,j}=\frac{y_{i}/x_{i}}{y_{i}/x_{i}}$$

where *α_i,j_* is the relative volatility, *y_i_* and *x_i_* are the molar ratios of the volatile component in gas phase and liquid phase and *y_j_* and *x_j_* are the molar ratios of water in gas phase and liquid phase, respectively.

However, as the development of membrane technology, some hybrid membranes have been used for separation of organic from the water mixture, which could be considered as the combination of VMD and pervaporation [93,94]. The selective layer functioning as pervaporation membrane normally contacts the feed, selectively concentrates/rejects organics into/from this layer and changes the molar ratios of the organic components to the water in comparison to that in the feed. The hydrophobic layer under the selective layer functioning as the MD membrane isolates the feed from the permeate and separates organics from water based on the Raoult’s law. Since the molar ratios of the organic components to the water at the interface between the selective layer and hydrophobic layer is altered, the separation factor of organics to water will differ from the common MD membrane.

The key to modelling approaches to the pervaporation/MD hybrid membrane is to obtain the separation factor of organics to water in the selective layer, in which the modelling of pervaporation membrane for mass transfer could be followed [95]. Since the evaporation of different components at the interface of the hydrophobic layer will still be driven by vapour pressure difference, it can be calculated by Dalton’s law [92]:

(32) $$\Delta P=y_{i}\cdot P_{total}-x_{i}P_{i}^{0}$$

where *P*^0^*_i_* and *P*^0^*_j_* are the saturated vapour pressures of volatiles and water at temperature *T*.

The thermal conductivity through the selective layer can be estimated by

(33) $$\lambda =\lambda_{f}\varepsilon +\lambda_{solid}\left(1-\varepsilon \right)$$

## 3. Modelling Approaches

### 3.1. Conventional Modelling Approaches

In the conventional approach, the modelling is based on the established mass transfer mechanisms through porous material and heat transfer in the fluid. Characterisation of the membrane and flow dynamics are critical to providing acceptable modelling results.

For all configurations, the data required for the modelling on the feed side include:

- membrane characteristics, based on Equations (14) and (15), including porosity, pore size, pore tortuosity, membrane thickness and thermal conductivity [22,96]. Furthermore, as the properties of some membranes will be changed under the operation conditions, such as being compressed under pressure, the correlations between the membrane properties and the operation conditions need to be set up [22,58,97];
- the configurations of the module, including the length and width of the flow channel, hydraulic diameter and spacer structure (Equations (8)–(11)); and
- parameters of the feed stream, including thermal conductivity, viscosity, linear velocity, salt concentration and temperatures.

The permeate side of the four MD configurations are distinct from each other and the information required is different.

- For DCMD, the information required is the same as the feed side.
- For AGMD, based on Equations (20) and (21), the information required is the air gap width, thermal conductivities of the air and condensate, the thickness of the condensate layer (which could be calculated based on the theoretical model), thickness and thermal conductivity of the cooling plate, and parameters of the cooling stream.
- In SGMD, the required seeping gas properties include thermal conductivity, viscosity, linear velocity, water content and temperatures.
- The permeate side of VMD is under reduced pressure. Therefore, pressure in the vacuum chamber is commonly the only parameter that needs to be known.

To simplify the modelling process, some assumptions are often proposed, although sometimes they is not stated in the literature. The following assumptions are generally used for all MD configurations [22,61,98,99]:

- One direction of flow;
- No heat loss from the module;
- No property variation along the whole piece of membrane;
- Only vapour phase in the pores;
- No convective heat transfer in the membrane pores;
- No influence of pore size distribution on mass transfer;
- Vapour pressure, velocity profile and temperature profile not being affected by the dissolved salt at low concentration; and
- Membrane pores being cylindrical and not interconnected.

In DCMD, no extra assumption is required for permeate flow, since it is also in liquid phase. The errors incurred by applying these assumptions into the modelling are normally in the range of ±5–10% [21,61,100], although systematic error could be caused mainly due to variation of the feed temperature. However, the feed temperature in MD is normally limited, in the range of 40–80 °C, which does not lead to the error of the predicted results greater than 10% of the experimental results.

On the permeate side of the AGMD, the assumptions related to the air gap and condensate on the cooling plate include [73,101,102,103]:

- No total pressure variation inside the air gap;
- Condensate in form of film, whose thickness is much thinner than that of the air gap; and
- Mass transfer mechanism dominated by diffusion.

The assumption of heat conduction or heat convection through the air gap (0.8–13 mm) and the condensate layer should not cause a big difference, since both thicknesses are small. The error incurred by that assumption is in the range of ±2–10%.

A sweeping gas is used in SGMD to strip vapour from the feed. It is commonly assumed that there is no vapour pressure gradient in the sweeping gas in the direction perpendicular to the flow direction of the sweeping gas [75,104]. The error caused by the assumption is in the range of ±2–12%.

The error of the experimental validation of the conventional modelling is generally less than 10% [61,105,106,107,108], which has been used to estimate the membrane area and operation parameters for in the pilot tests [91,109].

The conventional modelling approach does not require significant assistance from software and could provide reliable prediction as far as the MD system being characterised properly. However, this approach cannot show the profile of the temperature distribution in the stream and requires comprehensive characterisation work.

### 3.2. Computational Fluid Dynamics (CFD)

In CFD, the fluid flow is simulated numerically. Theoretically, CFD is still based on the conventional mass and heat transfer theory. Therefore, the fundamental characterisation work and assumption would be similar to the conventional modelling process. However, with the assistance of the embedded database/software package, CFD could simulate the three-dimensional temperature profiles and flux for the module with different spacers and/or geometric structures [23,110,111]. Hence, the optimum structure or flow dynamic condition can be achieved numerically. Although CFD will increase computational cost with increased geometric dimensions (2D to 3D) and resolution, it will save a lot of effort and cost in the fabrication of a real module or conducting real experiments [112]. Furthermore, CFD is able to present the local details, such as the temperatures and concentrations throughout the module, identify the dominant parameters hindering the mass transfer and achieve the optimisation of heat and mass transfers in the modules [21,99,113,114].

The experimental validation error of CFD is morally less than 5% under the tested conditions [21,113,115,116], which sets a fair confidence and a strong correlation between the model predictions and the experiments.

Besides the process optimisation, in comparison with the conventional modelling approaches, CFD can be used in geometry optimizations of module for all four MD configurations to achieve high-energy efficiency and flux with the assistance of required databases [110,113,117,118]. Therefore, the exiting databases or software packages are the keys for the development of CFD modelling. However, those packages are not developed specifically for MD, in which the great heat loss due to the latent heat transfer combined with mass transfer from the membrane surface might be different from the mass transfer or heat transfer process only.

### 3.3. Response Surface Methodology (RSM)

The RSM modelling approach is a visualisation technique based on mathematical statistics and can plot a 3D response surface with two variables [27,28,31,119]. In this approach, polynomial fits are conducted for sets of experimental data to form an adequate representation of the experimental response.

Since it is based on polynomial fits to sets of data from experiments, all the data recorded from the experiment can be used as both the variables and functions. For example, in DCMD, the flux can be fitted as functions of temperatures (outlet or inlet) and stream velocities and can be a variable for the function of the velocity or temperature, which is useful in commercialization. In the commercial operation, the output (flux or energy input) is set first and then the control parameters are figured out. These parameters can be calculated based on RSM.

RSM is based on the mathematic static of the experimental data to achieve the minimum Root Mean Square Error (RMSE). The error between the experimental and predicted fluxes is generally no greater than 0.1 (10%), depending on the coefficient of determination [120,121].

There quite a few advantages of RSM, which include:

- complicated characterizations of membrane properties and module parameters exist, such as measuring pore size, porosity, thickness and thermal conductivity of the membrane and determining the geometric structure of MD module, which are not as essential in conventional and CFD methods;
- variation of membrane properties do not affect the modelling results, since the recorded experimental data have contained the variations, which will be involved in the fitting process;
- control parameters can be predicted from the required output such as flux or energy requirement; and
- synergic effects of two variables on one function can be conducted for process optimisation.
- However, there are also some limitations of RSM:
- The modelling results from RSM may not be able to apply to other systems. The experimental data are foundational for RSM. The data used in the RSM normally include solute concentration, stream velocity, temperature, flux, GOR, vapour pressure and energy input [27,29,31,121,122]. However, all these data should be from the identical systems, and the predicted results should be strictly applicable to these identical systems. For example, if the spacer in the channel is replaced or a different type of membrane is used, the response of the modelling might be altered.
- A comprehensive amount of experimental work/variables is required to achieve the representative data and minimise the error. The results predicted by RSM would be more accurate if the increased data and parameters acquired from the experiment are used for the fitting process.
- It is not possible to simulate the data that can be measured or calculated based on the measurement data. For example, *TPC* cannot be calculated by the measurable data and cannot be predicted by the RSM. However, Cheng et al. overcame this limitation by combining conventional modelling with RSM [31].

Theoretically, no assumption is required in RSM, which is quite different from the other two modelling approaches, because the fitting procedure of the real experimental data in RSM has involved all the factors that might lead to the results.

### 3.4. Critical Assessment and Comparison of the Different Approaches

There is no perfect modelling approach for all conditions as far as the authors know, but there should be a best approach for each specific situation, depending on the aims of the modelling. In Table 3, a summary of the research publications related to the three approaches and advantages and disadvantage of the three approaches are shown.

It can be found that the conventional modelling approach is still the most popular one since it is based on classic theories on mass transfer and heat transfer phenomena and is fundamental for CFD, although some semi-empirical equations are used [133,134]. Characterisation of membrane, module and process parameters are required for the modelling. Under the most situation, assumptions are used to simplify the model. Since the conventional modelling is focused on the mass and heat transfers across the membrane, it is the foundation of all other modelling approaches. It would not be possible to employ other modelling approaches efficiently without understanding the conventional modelling. The conventional modelling approach is a good tool for assessment of and optimisation of the operation parameters of an existing system with a similar module structure to achieve high flux or thermal efficiency. However, it is the lack of the ability to predict the temperature profile and concentration profile in three dimensions that is significant for the module design. Furthermore, since it focuses on what occurs through the membrane, it could not be used for the assessment and optimisation of the overall system.

CFD is a very versatile modelling approach, and it employs computer science and allows users to adjust more parameters and observe local transfer phenomena with the available software packages, which are programmed based on the classic theories. It can do all the jobs that conventional modelling is able to do and figure out the temperature and concentration profiles in three dimensions to locate the stagnant section in the module. Based on these capabilities, CFD can be used to optimise the structure of the flow channels to minimise the concentration and temperature polarisations and facilitate the module design, especially multistage module design, which could make MD competitive to other commercialised separation. It also can be found in Table 3 that most of the papers have focused on the influence of enhanced stream turbulence on MD performance by varying geometry of spacers and flow channel structure/design. However, most CFD research studies focused on oversimplified module geometry or physical phenomena currently [21]. With the assistance of computer technology, CFD has the ability to present detailed three-dimensional profiles of the targeted parameters in a module with complex structures and during the development of fouling caused by settlement of suspended solid and crystallisation, and development of membrane wetting incurred by concentration polarisation, fouling and surfactants. CFD modelling of those phenomena is significant in MD commercialisation and should be developed in future studies.

The RSM approach is the least used approach in research, although it seems very versatile. It can be used for the overall assessment of an existing system based on the data acquisition by collecting data from experiments. In RSM, all the parameters of the system can be used as either variables or functions, which are different from the above modelling approaches. For example, flux and/or operational cost could be used as the variables for a function of feed inlet temperature in RSM. When the productivity or operational cost of a given system is defined, the feed inlet temperature could be estimated to achieve the targets. This method does not require researchers to know heat and mass transfer phenomena in MD, but it does require the researchers to have sound mathematic skills. Similar to the conventional model, RSM cannot be used to look at the local phenomena or for the optimisation of a module structure. Since it is data-based and does not rely on the characteristics shared by all MD processes, the outcomes from RSM are only valid for the system where the data are collected, which could be a key hinderance for wide employment of RSM.

### 3.5. Modelling of Operational Cost of MD

All the reviewed modelling approaches can be used to assess the thermal energy cost and electrical cost based on the operational conditions. However, those models cannot include the cost incurred from investment, maintenance and pretreatment. Since there are not many full-scale MD plants reported [135], the cost estimation cannot represent the real cost.

As far as the authors know, there are only a few full-scale MD plants operating in China [20]. From the best practice of those plants, it was found that the overall cost is less by using steam than by using 80 °C hot water as heat sources. It could be varied with the conditions available when estimating the cost. However, it is obvious that when high-grade heat is used, less membrane area, module size, footprint and operational time are required for the same treatment capacity of the plants, which was hardly shown in the cost estimation.

It was also reported by Kesieme, et al. [136] that the electrical cost and pretreatment cost of MD is close to 30% and 60% of that of the reverse osmosis system, which requires no thermal energy. Furthermore, with the increase of energy efficiency in the entire industry, the free thermal energy currently could be as low as 40 °C [65]. To achieve the same productivity, significantly more membrane area or pumping energy would be required. Therefore, it is worth considering the modelling of MD employment in some areas where conventional membrane processes are hard to handle, such as highly acidic wastewater being treated by the full-scale plant operating in China.

## 4. Research Gaps and Future Study

There is presently a gap between the development of a theoretical transfer mechanism and modelling approaches and the emergence of new types of membranes with new frameworks or incorporated with functional particles [90,137,138,139,140]. With the development of novel membranes, MD can currently be employed in some areas where it was not possible to use it previously, such as volatile organics removal and treatment of surfactant-containing wastewater [25,90,91,141,142]. The fundamental mechanism at the molecular scale has not been identified to explain why the addition of the metal organic framework is able to enhance the membrane flux [140], how the dense-layer coating stops the membrane wetting from surfactants and organics or in what form (vapour, liquid or as molecule) the water or organics transport across the dense layer [90]. Zhao et al. [143] developed a model using a conventional method for optimisation of the hydrophilic/hydrophobic dual-layer membrane. However, the mass transfer resistance in the hydrophilic layer was ignored based on the structure of the membrane. Furthermore, most of the membrane modification work using inorganic particles claims that the porous structures or micro channels in the materials facilitate the mass transfer [140], but it has not been proven that those channels would still allow water molecules to pass through after the particles are embedded in the polymer.

There is also a gap between the cost analysis of commercialisation, including investment, total energy consumption, maintenance cost and operation cost, and cost modelling. Currently, the models only focus on the thermal energy consumption/energy efficiency of MD and achieve a very competitive operation cost of MD to other processes, which have misled the MD commercialisation in some full-scale plants.

Hence, future studies should work on the mass transfer mechanism and modelling of MD membrane with multiple functional layers and cost analysis of MD for the barriers to commercialisation, which would determine the fate of MD development.

## 5. Conclusions

As another distillation process, simultaneous heat and mass transfers also occur in MD. To reduce thermal energy consumption, multistage configuration would be an effective measure. Temperature polarisation could be used as a parameter for assessment of the MD process, but low-temperature polarisation is not directly linked to the high thermal efficiency and high flux.

The TPC is not feasible to be used for the assessment of driving force polarisation. The rate of driving force is developed in this paper to fulfil the gap.

Transfer phenomena in four major configurations of MD and three major modelling approaches, including conventional modelling, CFD and RSM, have been briefly discussed in this paper. A summary of assumption for the various approaches has been made.

VMD has the highest thermal energy efficiency and flux in all four configurations, and its mass transfer mechanism is *P*–*K* transition, in which both Poiseuille flow and Knudsen diffusion contribute to the mass transfer. The mass transfer mechanism in all other configurations is *K*-*M* diffusion transition, in which Knudsen diffusion and molecular diffusion are connected in series.

Conventional modelling is based on classic mass transfer and heat transfer theory and is the foundation of the CFD. However, by utilising the available database or software package, CFD can predict the influence of geometry change on MD performance and conduct module structure optimisation.

RSM is a visualization technique based on mathematical statistics. It can be set up with sets of experimental data and will not be affected by the property variation under experimental conditions such as those of conventional and CFD modelling. In combination with the conventional modelling approach, RSM can be used to predict the parameters that cannot be measured or calculated by the measurement data, such as *TPC*.

Transfer phenomena of the emerging MD membrane need to be studied and modelled to provide guidance for membrane modification.

A comprehensive cost study would be significant for MD commercialisation.

## Figures and Tables

**Table 1:** Mass transfer principles of gas through porous membrane [51].

State in the Pore			*Kn* < 0.01	0.01 < *Kn* < 1	*Kn* > 1
Composition	Total Pressure Difference	Partial Pressure Difference			
Gas mixture	0	≠0	M	K-M	K
Single gas	≠0	N/A	P	P-K	K
Gas mixture	≠0	≠0	P-M	P-M-K	K

**Table 2:** Reported flux and thermal efficiency of different single-stage MD configurations.

	DCMD	AGMD	SGMD	VMD
Water Flux (Lm^−2^h^−1^)	7–55 [80]	0.5–7 [81]	4.3–23 [14]	13.9–56.2 [82,83]
Thermal efficiency (%)	20–70 [17,84]	90–98 [85]	30–95 [60,86]	90–98 [87,88,89]

**Table 3:** Summary of research publication of different approaches in 2015–2020.

Approaches	Published Articles	Classification Based on Approach Method	Applications	Merits	Disadvantages/Limitations
Conventional modelling	~8000	Conventional heat and mass transfers	Prediction of performance influenced by feed properties, operation conditions, membrane characteristics, configurations [73,123,124,125,126]	Assists comprehensively understanding transfer phenomena Prediction trends/results are generally applicable to different systems	Time-consuming characterisation of module configurations, membrane, operation conditions Systematic errors could occur due to assumptions, although within the range of ±5% in the modelling range
CFD	~1000	Computer science	Prediction of performance influenced by geometric structure of turbulence promoters (spacer baffle plate), modules structure [23,26,110,111,127,128]	Assists understanding of local transfer phenomena Assists optimisations of module and flow channel Fancy animation could be used to simulate the experimental	Computer science technology is required Time-consuming characterisation is also required Theories for mass and heat transfers in the conventional modelling could be required depending on the availability of the software packages
RSM	~700	Data acquisition from the overall system	Prediction of performance influenced by fouling and wetting, membrane characteristics, operation conditions, module geometric dimensions, feed properties [120,129,130,131,132]	Assists overall assessment of a given system in the respects of feed properties, operations conditions, and module configurations Characterisation of systems are not essential Mechanism of heat and mass transfers and assumptions are not essential	Predicted results are theoretically confined to the modelled system

## Data Availability

Data available in a publicly accessible repository.

## Nomenclature

*A*	membrane area
∆*Q_f_*	heat loss from the feed flow
*A_cross_*	cross sectional area of empty channel
*b*	membrane thickness
*b_air_*	thickness of the air gap
*C_f_*	specific heat of the feed
*d*	pore diameter
*D_AB_*	relative diffusivity of the vapour (A) to air (B)
*d_f_*	diameter of spacer filaments
*d_h_*	hydraulic diameter of the spacer filled channel
*E*	thermal efficiency
*H_latent_*	latent heat of vaporization
*h_sg_*	convective heat transfer coefficient
*J*	flux
*J_m_ and J_k_*	vapour fluxes contributed, respectively, by molecular and Knudsen diffusions
*k_B_*	Boltzman constant
*Kn*	Knudsen number
*l*	mean free path
*l_m_*	mesh size
*ṁ* *_f_*	mass flow rate of the feed
*m_w_ and m_a_*	molecular weights of vapour and air
*P* ^0^ *_i_* *, and P* ^0^ *_j_*	saturated vapour pressures of volatiles and water
*P_g_ and T_g_*	mean pressure and temperature in the air gap
*P_pore_*	pressure in the pore
*P_T_*_1_ * and P_T_*_2_	vapour pressures on feed and permeate sides at membrane interfaces
*Q*_1_ *,Q*_2_	total heat transferred across the membrane
*Q_f_*	volumetric flow rate of feed
*Q_sg_*	heat transfer from the membrane surface on the permeate side to the bulk gas stream
*Q_vacuum_*	heat transfer on permeate side in VMD
*R*	universal gas constant
*R_m_ and R_k_*	mass transfer resistances to molecular and Knudsen diffusions
*S_spacer_*	specific surface of the spacer
*t*	pore tortuosity
*T*	mean temperature in the pores,
*T*_1_ * and T*_2_	temperatures at the membrane surfaces of the feed and permeate sides
*T_c_*	surface temperature of the condensate
*T_cl_*	temperature of interface between the cooling plate and the cooling flow
*T_cl,b_*	temperature of the bulky cooling flow
*T_f,i_ and T_f,o_*	inlet and outlet temperatures of feed
*T_pl_*	temperature of interface between the condensate and the cooling plate
*T_sg_*	temperature of the bulk gas flow
*v_s_*	velocity in the spacer filled channel
*x*_2_ * and x_c_*	molar fractions of the water vapour at surfaces of membrane and condensate
*y_i_ and x_i_*	molar ratios of the volatile component in gas phase and liquid phase
*y_j_ and x_j_*	molar ratios of water in gas phase and liquid phase
*α*	convective heat transfer coefficient
*α_f_*	the convective heat transfer coefficient
*α_i,j_*	relative volatility
*ε*	porosity of membrane
*ε_spacer_*	porosity of spacer
*λ, λ_air_, λ_f_ and λ_solid_*	thermal conductivities of membrane, air, feed and the membrane material
*λ_c_ and b_c_*	thermal conductivity and thickness of the condensate layer
*λ_pl_ and b_pl_*	thermal conductivity and thickness of the cooling plate
*μ µ_g_*	viscosities of liquid and gas
*ρ*	density of the liquid steam
*σ*_1_ * and σ_a_*	collision diameters of vapour and air
*τ*	temperature polarisation coefficient
